# Nitrogen-based gas molecule adsorption of monolayer phosphorene under metal functionalization

**DOI:** 10.1038/s41598-019-48953-0

**Published:** 2019-08-29

**Authors:** Shuangying Lei, Ran Gao, Xiaolong Sun, Sijia Guo, Hong Yu, Neng Wan, Feng Xu, Jie Chen

**Affiliations:** Key Laboratory of Microelectro mechanical Systems of the Ministry of Education, Southeast University, Nanjing, 210096 China

**Keywords:** Two-dimensional materials, Materials science

## Abstract

Using the first-principles calculation based on density functional theory (DFT), the adsorption properties of nitrogen-based gases molecules (NH_3_, NO, NO_2_) on various metal (Li, Na, K, Rb, Cs, Ca, Sr, Ba, Ni, La, Tl) decorated phosphorene systems have been studied systematically. The results show that all metal decorations can improve the adsorption strength of phosphorene to nitrogen-based gases molecules except for Tl decoration. Especially, the adsorption energy of NH_3_ molecule on Ni decorated phosphorene is 1.305 eV, and the adsorption energies of NO and NO_2_ on La decorated phosphorene can be up to 2.475 and 3.734 eV, respectively. In addition, after NO and NO_2_ adsorptions, the electronic and magnetic properties of some metal decorated phosphorenes change, indicating that the metal decorated phosphorenes have great potential in NO and NO_2_ detection.

## Introduction

Nowadays, many human activities, such as burning, motor vehicle exhaust, and chemical production processes, produce polluting gases. Among many atmospheric pollutants, nitrogen-based gases (NO, NO_2_, NH_3_) have received widespread attention in recent years due to their threat to the ecological environment (acid rain, ozone depletion, photochemical smog) and human health^1–5^. Thus, the removal and detection of nitrogen-based exhaust gases from environment is essential. Traditionally, metal oxide semiconductors have been widely used to detect of nitrogen-based gases due to low cost, low power consumption and simplicity in measurements^6–9^. However, their long recovery period and low specificity limit the application in high measurement accuracy gas detection^10^.

The emergence of nanotechnology, especially the successful stripping of graphene^11,12^, opens up new avenues for gas detection. The large surface-to-volume ratio makes it be a good candidate for gas detection and removal^13–15^. The excellent gas detection capabilities of graphene were demonstrated, and the results showed that the sensitivity can reach a single gas molecule^16^. However, theoretical studies found that the interaction between pristine graphene and small molecule gases is rather weak^17^, and the adsorption strength of gas molecules can be improved by introducing decoration or doping atoms into graphene^18,19^. Recently, another single element 2D material, phosphorene, was successfully obtained by mechanical stripping methods. Unlike C atoms with inherent inertness, the chemically active of the phosphorus atom makes the phosphorene have stronger interaction with the atoms or molecules than graphene^20–24^. Especially for gas molecules, the studies indicate that phosphorene has the potential to compete with or even exceed graphene in gas adsorption and detection^22,23^. For example, Mayorga-Martinez *et al*. studied the sensing of methanol molecules based on a two-dimensional phosphorene sensor, and the results showed that the sensitivity can reach 28 ppm^25^. The experimental results by Abbas *et al*. showed that the detection sensitivity of NO_2_ down to 5 ppb^26^. Furthermore, the puckered honeycomb structure of phosphorene provides more adsorption space for adsorbate than the planar structure of graphene. Based on surface adsorption, the researchers studied the application of phosphorene in Na^+^ and Li^+^ batteries, and the results showed that the capacity found to be ~400 and ~700 mAhg^−1^ for Na and Li batteries^27–30^, respectively. Additionally, other applications based on surface decoration, such as hydrogen storage^31,32^ and the removal of polluting gases^33–35^, have been extensively theoretically investigated, and the results showed that the decoration of atoms can greatly improve the adsorption capacity and strength of phosphorene to the gas molecules.

In this paper, the behavior of nitrogen-based gas molecules adsorption on pristine and various metal (Li, Na, K, Rb, Cs, Ca, Sr, Ba, Ni, La, Tl) decorated phosphorene were studied systematically. It is found that all metal decorations can improve the adsorption strength of nitrogen-based gas molecules on phosphorene, except for NO molecule on Tl decorated phosphorene, and thus metal decorated phosphorenes have great potential for application in nitrogen-based gas removal. Furthermore, the electrical and magnetic properties of some metal decorated phosphorene have interesting change after NO and NO_2_ adsorption, indicating that metal decorated phosphorenes have great potential in NO and NO_2_ detection.

## Computational Methods

All the density functional theory (DFT) calculation have been implemented by using the Vienna ab initio simulation package (VASP)^36,37^. The generalized gradient approximation (GGA) with the parametrization of Perdew-Burke-Ernzerhof (PBE) was adopted^38^. The empirical correction scheme of Grimme (DFT + D2) was included to correct the effective van der Waals (vdW) interaction^39^. The kinetic energy cutoff was set to be 500 eV for all calculations. The 3 × 3 × 1 mesh grid was sampled in 1^st^ Brillouin zone by using Monkhorst-Pack scheme. To avoid the interaction between periodic adsorbates, the 3 × 4 supercells were employed. A vacuum of 15 Å was used perpendicular to the phosphorene layer to ensure that the adjacent phosphorene sheets do not interact with each other. For structural optimization, the force convergence criterion on each atom in the structure is less than 0.01 eV/Å. The adsorption energies (*E*_*ads*_) of nitrogen-based gas molecules on pristine phosphorene (bP) and metal decorated phosphorene (bP-M) are calculated by

1 $${E}_{ads}={E}_{total}-({E}_{bP/bP-M}+{E}_{adsorbate})$$

where *E*_*total*_ is the total energy of the adsorption system. *E*_*bP*−*M*_ and Eadsorbate are the energies of bP/bP-M and nitrogen-based gas molecule, respectively.

## Results and Discussion

### Nitrogen-based gas molecules adsorption on pristine phosphorene

**Figure 1:**
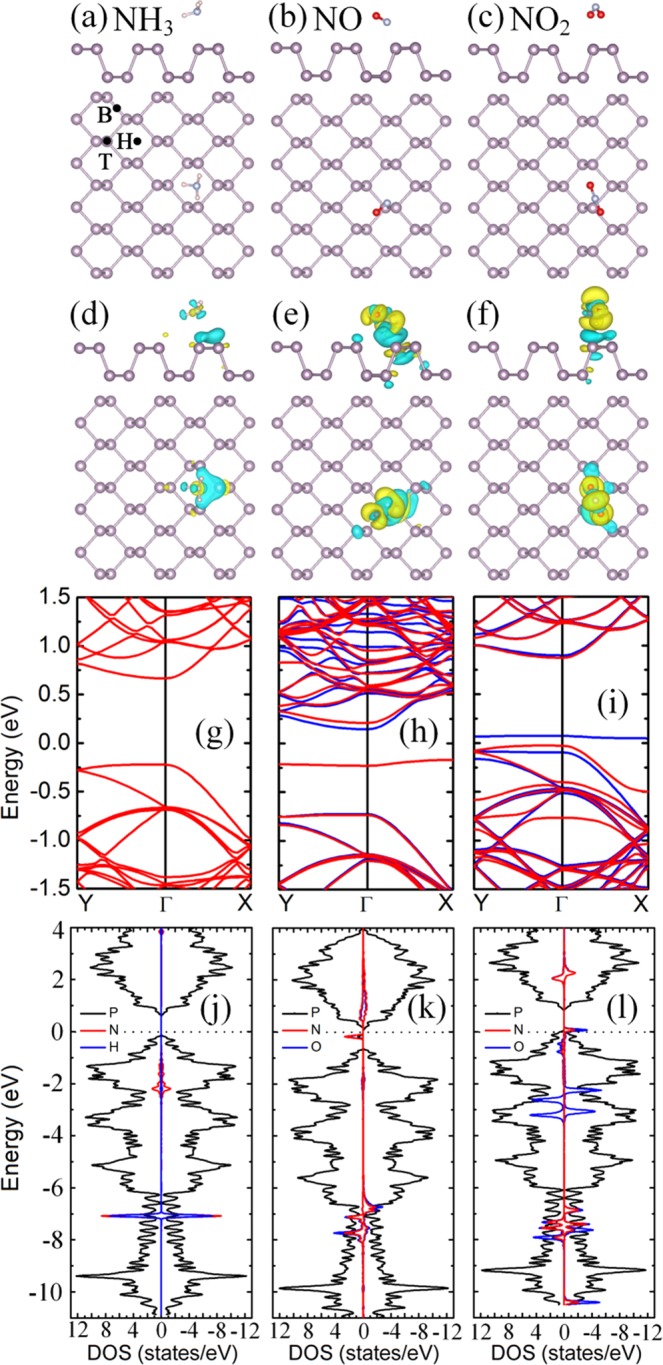
The optimized structure of (**a**) NH_3_, (**b**) NO and (**c**) NO_2_ adsorbed on 3 × 4 pristine phosphorene, the corresponding DCD (**d**–**f**), band structures (**g–i**), and LDOS (**j–l**), respectively. Purple, grey, pink, and red balls in (**a–f**) represent P, N, H and O atoms, respectively. Yellow and blue regions in (**d–f**) denote charge accumulation and charge depletion, respectively. The red and blue curves in (**g–i**) represent the spin-up and spin-down band, respectively. The black, red and blue curves in (**j-l**) represent the LDOS of P, N and H (O), respectively, and the Fermi level is set to zero in (**g–l**).

The optimized lattice constants along the armchair and the zigzag direction are 13.722 and 13.225 Å, respectively, and the bandgap is 0.88 eV for 3 × 4 monolayer phosphorene supercell with 48 phosphorus atoms, which are consistent with the ref.^40^. For the nitrogen-based gas molecules (NH_3_, NO, NO_2_) on pristine phosphorene, we consider three highly symmetric adsorption sites (hollow, bridge, top, as shown in Fig. 1(a)) and various initial adsorption configurations. After geometry optimization, the most stable adsorption configurations are obtained and shown in Fig. 1(a–c). The nearest atom-to-atom distances between the NH_3_, NO and NO_2_ molecules and the phosphorenes are 3.192, 2.216 and 2.714 Å, respectively, and the corresponding adsorption energies are 0.212, 0.250 and 0.220 eV. This means the smaller the distances, the larger the adsorption energies. The trend of the distances and adsorption energies are consistent with refs^22,23^, and the specific differences between data may be attributed difference exchange-correction functional. The valence radii are 1.07, 0.31, 0.71 and 0.66 for P, H, N and O atoms^21^. The nearest H-P distance of 3.192, N-P distance of 2.216 and O-P distance of 2.714 Å are larger than the sums of corresponding atomic valence radii, indicating that the adsorptions of NH_3_, NO and NO_2_ on phosphorenes are physical adsorptions. The slices of charge densities in Fig. S5 also corroborate physical adsorption characters. The NO molecule prefers to adsorb on the top site and its N atom point to the surface of phosphorene. The N-O bond length is 1.185 Å, which is slightly larger than that of 1.169 Å for the free NO molecule, due to the interaction between NO molecule and phosphorene. However, the NH_3_ and NO_2_ molecules prefer to adsorb on the hollow site. To analyze the intrinsic mechanism of the interaction between nitrogen-based gas molecules and phosphorene, the differential charge density (DCD) of the adsorption systems were calculated as shown in Fig. 1(d–f). There is electron transfer between nitrogen-based gas molecules and phosphorene. The charge transfer quantities between the NO/NO_2_ molecules and phosphorenes are larger as compared with NH_3_ adsorbed case, implying the larger interaction between the NO/NO_2_ molecules and phosphorenes and thus larger adsorption energies. The charge transfer quantity between the NH_3_ molecule and phosphorene is very small, indicating that the interaction between NH_3_ and phosphorene is weak, which is consistent with the minimum adsorption energy. In addition, the Bader charge analysis indicates that the electrons transfer from phosphorene to NO and NO_2_ molecules are 0.242 and 0.300 e, respectively, while the electrons transfer from NH_3_ molecule to the phosphorene is 0.030 e. Thus, the NO and NO_2_ molecules act as acceptors, whereas the NH_3_ molecule acts as a donor, which is consistent with ref.^22^.

To understand the effects of the adsorption of NO, NO_2_ and NH_3_ molecules on the electronic properties of phosphorene, the band structures and local density of states (LDOSs) of the adsorption systems were calculated, as shown in Fig. 1(g–l). The adsorption of NO and NO_2_ molecules introduce spin polarization in phosphorene, while the NH_3_ adsorption system is of zero magnetic moment (see Table 1). In addition, both the conduction band minimum (CBM) and valence band maximum (VBM) for the three adsorption systems remain at the Γ-point. Thus, the adsorption of nitrogen-based gas molecules does not change the direct-bandgap-semiconductor properties of phosphorene, and the corresponding bandgaps are 0.888, 0.869 and 0.899 eV, respectively. For NH_3_ adsorbed case, as shown in Fig. 1(j), the states of NH_3_ molecule are far from Fermi level, and thus adsorption of NH_3_ molecule doesn’t change the bandgap of phosphorene. Interestingly, there is a flat band near the Fermi level for the NO and NO_2_ molecular adsorption systems, which correspond to the sharp peaks near Fermi level in LDOSs. For NO adsorbed case, strong hybridization of the states between NO molecule and phosphorene near Fermi level is responsible for large interaction and the largest adsorption energy (see Fig. 1(k)). On basis of the definition in ref.^41^, the channel of phosphorene with NO adsorption is narrower than that of pristine phosphorene by 0.05 Å, resulting in the stronger repulsive interaction between the facing lone pairs at the ditch, which should be mainly responsible for the decrease of bandgap. For NO_2_ adsorbed case, as shown in Fig. 1(l), the coupling peaks near the Fermi levels is mainly arise from NO_2_ molecule. Additionally, there are two O peaks near VBM, which is responsible for the large adsorption energy. The charge transfer quantity of 0.3 e from phosphorene to NO_2_ molecule reduces the lone pair electrons and thus reduces repulsive interaction between the facing lone pairs at the ditch, which leads to the larger bandgap of 0.899 eV^42^.

**Table 1:** The charge transfer amounts (*∆Q*), adsorption distances (*D*_P-Gas/M-Gas_) between the nearest P/metal atoms and nitrogen-based gas molecules, magnetic moments (*M*) and adsorption energies (*E*_*ads*_) for nitrogen-based gas molecules adsorption on pristine/metal decorated phosphorene.

Metal	*∆Q* (e)			*D*_P-Gas/M-Gas_ (Å)			*M* (μB)			*E*_*ads*_ (ev)		
	NH_3_	NO	NO_2_	NH_3_	NO	NO_2_	NH_3_	NO	NO_2_	NH_3_	NO	NO_2_
bP	0.030	−0.242	−0.300	3.192	2.216	2.714	0.000	1.000	0.902	0.212	0.250	0.220
Li	−0.022	−0.466	−0.906	2.012	1.910	1.932	0.000	1.507	0.000	1.079	0.831	2.534
Na	−0.009	−0.398	−0.841	2.404	2.314	2.280	0.000	1.385	0.000	0.821	0.647	2.202
K	0.031	−0.340	−0.708	2.880	2.710	2.647	0.000	1.360	0.166	0.546	0.519	1.823
Rb	0.027	−0.330	−0.692	3.060	2.886	2.791	0.000	1.353	0.192	0.478	0.476	1.738
Cs	0.023	−0.313	−0.664	3.260	3.143	3.010	0.000	1.343	0.240	0.476	0.466	1.705
Ca	0.013	−0.605	−0.802	2.494	2.180	2.310	0.000	1.670	0.000	1.07	1.538	3.485
Sr	−0.005	−0.605	−0.824	2.708	2.348	2.474	0.000	1.679	0.000	0.917	1.363	3.337
Ba	−0.008	−0.569	−0.822	2.841	2.521	2.660	0.000	1.705	0.000	1.066	1.339	3.14
Ni	0.118	−0.332	−0.557	2.034	1.676	1.895	0.000	1.000	1.000	1.305	2.121	1.818
La	0.022	−0.711	−0.770	2.703	2.172	2.469	0.990	0.653	0.000	1.185	2.475	3.734
Tl	0.034	−0.190	−0.717	3.150	2.815	2.575	0.000	1.458	0.000	0.238	0.252	1.773

Positive *∆Q* denotes electron transfer from nitrogen-based gas molecules to pristine/metal decorated substrates.

### Metal atoms decorated pristine phosphorene

**Figure 2:**
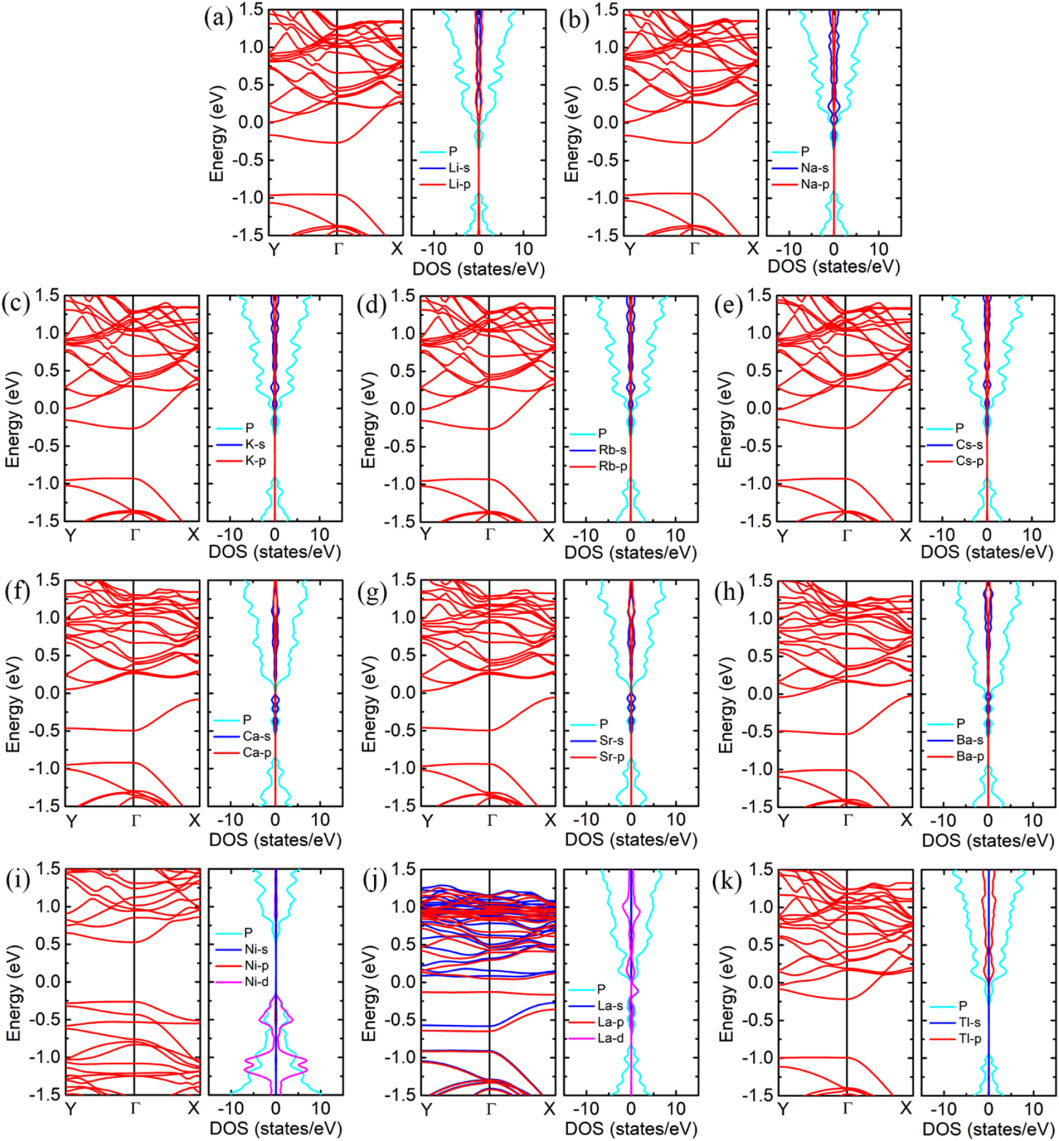
The band structures and PDOSs of (**a**) bP-Li, (**b**) bP-Na, (**c**) bP-K, (**d**) bP-Rb, (**e**) bP-Cs, (**f**) bP-Ca, (**g**) bP-Sr, (**h**) bP-Ba, (**i**) bP-Ni, (**j**) bP-La and (**k**) bP-Tl. The red and blue curves in the band structures represent the spin-up and spin-down bands, respectively. The cyan curves in PDOS represent the states for P atoms, and the blue, red and magenta curves represent the *s-*, *p-* and *d-*states for metal atoms.

To further improve the adsorption of phosphorene to nitrogen-based gas molecules, metal atoms are employed to decorate the phosphorene. Considering the growth morphology of metal atom on the surface of the phosphorene, and to avoid the formation of metal clusters, eleven metals with adsorption energy larger than bulk cohesive energy were selected out for decoration aims, namely alkali metal (AM = Li, Na, K, Rb, Cs), alkaline earth metal (AEM = Ca, Sr, Ba), transition metal (TM = Ni, La) and post transition metal (PTM = Tl)^43^. Moreover, the electronic structures of the bP-M systems are shown in Fig. 2 for comparison. Due to the transfer of electrons between the metal atoms and phosphorene, the Fermi levels of phosphorene have different degrees of shifting. Except for bP-Ni, the other metal electronic states distribute within the conduction bands of phosphorene. This leads to that electrons of metals are transferred to phosphorene and, correspondingly, the Fermi levels of phosphorene shift upward^21,41^. Thus, the phosphorene undergoes semiconductor-to-metal transition, due to upward shift of Fermi level, after AM, Ba and Tl decorations. For the bP-Ca, bP-Ca and bP-La systems, on the other hand, the lowest conduction bands of phosphorenes are pushed down relative to the other conduction bands due to the repulsive interaction of metal states. The spacings between the lowest and the second lowest conduction bands are of different degrees of increasing, especially for the AEM and La decorations cases^42^. When the Fermi levels shift upward and are located in the large spacings between the lowest and the second lowest conduction bands, the bP-Ca, bP-Ca and bP-La systems exhibit small indirect bandgaps. For the bP-Ni system, the states are mainly distributed in valence bands, leading to small amounts of electron transfer. Thus, the bP-Ni system remains the direct-bandgap-semiconductor property with band gap of 0.788 eV. The decrease of bandgap for the the bP-Ni system may be attributed to introduction of states near VBM. Additionally, the decoration of La atom causes the phosphorene to exhibit magnetism.

### NH_3_ gas molecule adsorption on bP-M

**Figure 3:**
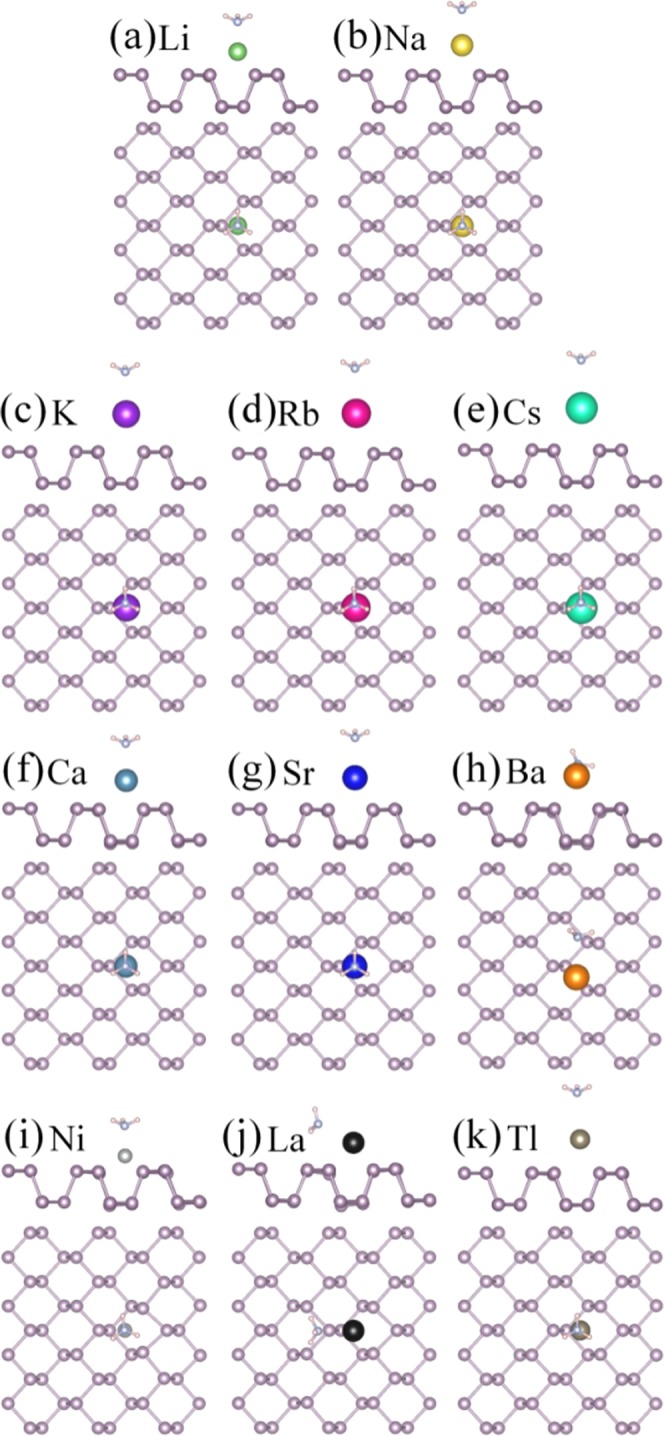
The optimized structures of NH_3_ adsorption on bP-M (M = Li, Na, K, Rb, Cs, Ca, Sr, Ba, Ni, La, Tl). The green, gold, purple, red, cyan, dark cyan, blue, orange, light gray, black and gray balls represent Li, Na, K, Rb, Cs, Ca, Sr, Ba, Ni, La and Tl, respectively.

For the NH_3_ molecules adsorption on bP-M systems, the various initial adsorption configurations are considered. The most stable adsorption configurations are obtained by comparing the adsorption energies after full geometry optimization. The most stable adsorption configurations are illustrated in Fig. 3, and their corresponding adsorption energies (*E*_*ads*_) are listed in Table 1. All NH_3_ molecules are stably adsorbed on bP-M systems with N atoms pointing toward the metal. The adsorption energies range from 0.238 to 1.305 eV for all the stable adsorbed cases, which are larger than that of 0.218 eV for adsorption on pristine phosphorene, indicating that the decoration of metal can improve the adsorption energy of phosphorene to NH_3_ molecule. In addition, the charge transfer amounts (*∆Q*) and adsorption distances (*D*_P-Gas/M-Gas_) between NH_3_ and metal decorated phosphorene are also summarized in Table 1. The largest adsorption energy and the largest charge transfer amount indicate the strongest adsorption of bP-Ni to NH_3_.

**Figure 4:**
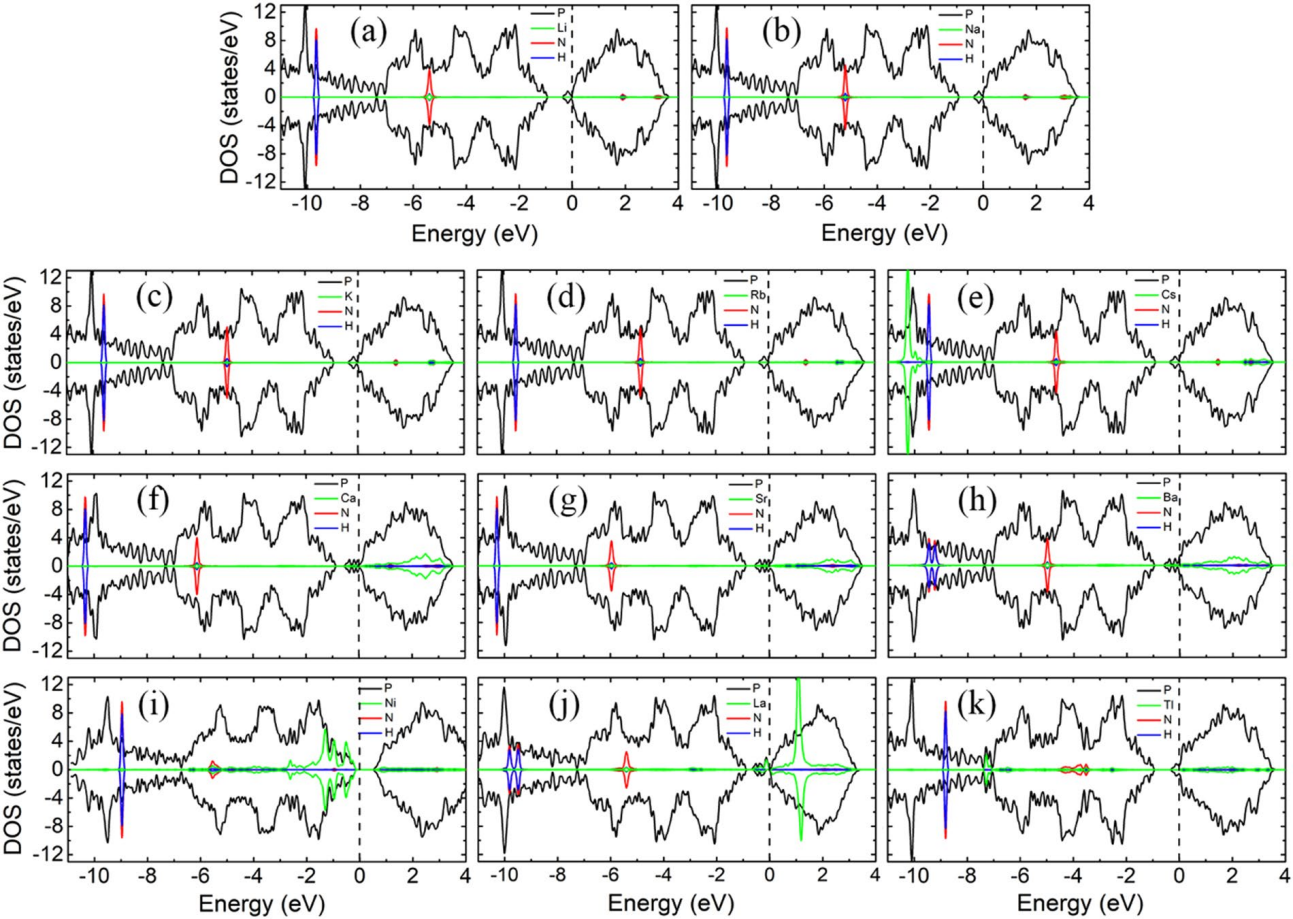
The LDOSs of NH_3_ adsorption on bP-M (M = Li, Na, K, Rb, Cs, Ca, Sr, Ba, Ni, La, Tl). The black, green, red and blue curves represent the LDOS of P, metal, N and H, respectively. The Fermi level is set to zero.

Figure 4 shows the LDOS of NH_3_ molecules adsorption on bP-M systems. The adsorptions of NH_3_ molecules have little effect on the electronic structures of bP-Ms due to that the states of N and H atoms are far below the Fermi level. As shown in Fig. 4(j), the asymmetric LDOS distribution of bP-La adsorption system indicates that bP-La adsorption system is spin-polarized with magnetic moment of 0.990 μB, while the other bP-Ms adsorption systems have no spin polarization. The magnetic properties of bP-Ms with NH_3_ adsorption are consistent with those without NH_3_ adsorption, which are also corroborated by the band structures in Fig. S7, implying that NH_3_ adsorption doesn’t change magnetic property of bP-M adsorption systems.

The Fermi levels of the decorated substrates shift slightly due to the certain amount of electron transfer between the decorated substrates and NH_3_ molecules. Except for bP-Sr, the conductive properties of the other bP-M system remain unchanged after NH_3_ adsorptions. For example, the bP-AM (AM = Li, Na, K, Rb, Cs), bP-Ba and bP-Tl adsorption systems still remain metal properties, while the bP-Ca, bP-Ni and bP-La adsorption systems still remain semiconductor properties with the corresponding bandgaps of 0.045, 0.788, and 0.188 eV, respectively. For bP-Sr adsorption system, as shown in Figs 4(g) and S7(g), the Fermi level enters the second lowest conduction band of phosphorene, indicating that the bP-Sr undergoes semiconductor-to-metal transition after NH_3_ adsorption. In addition, the overlap between the coupling peaks of N and H and the metal indicates strong hybridized interaction between them, which may be the cause that the metal decoration can improve the adsorption strength of phosphorene to NH_3_ molecules. However, for the NH_3_ molecule adsorption on the bP-Tl system, except for the N and H coupling peaks near −4 eV in Fig. 4(k), the N and H coupling peaks are not significantly associated with Tl LDOS, indicating the weak interaction between the NH_3_ molecule and T1 atom.

### NO gas molecules adsorption on bP-M

**Figure 5:**
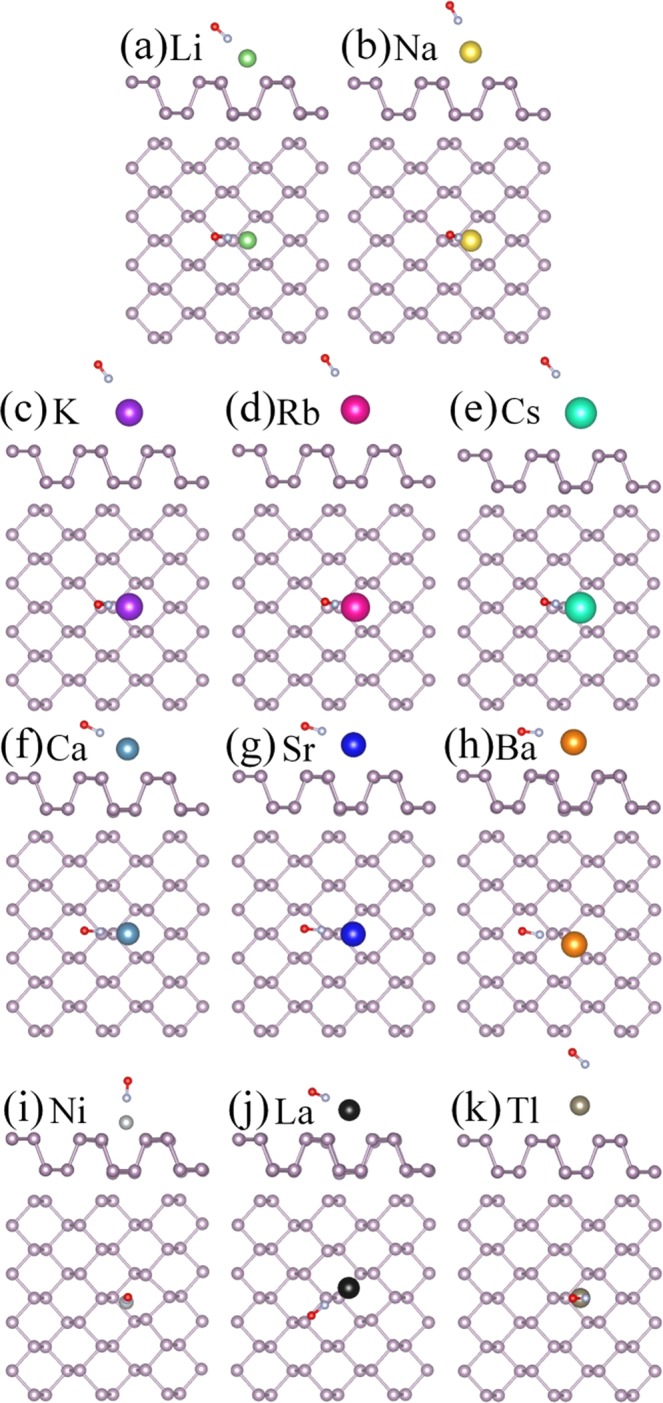
The optimized structures of NO adsorption on bP-M (M = Li, Na, K, Rb, Cs, Ca, Sr, Ba, Ni, La, Tl). The green, gold, purple, red, cyan, dark cyan, blue, orange, light gray, black and gray balls represent Li, Na, K, Rb, Cs, Ca, Sr, Ba, Ni, La and Tl, respectively.

Three different adsorption configurations were modeled for NO adsorption on bP-M: the N-O bond is parallel to phosphorene plane and N-O bond is normal to phosphorus plane with N or O pointing to metal atom. The results show that the NO molecule prefers the N atom to be close to the metal atom due to the larger adsorption energy, as shown in Fig. 5. The adsorption energies were calculated and summarized in Table 1. For the NO molecule adsorptions on bP-AM systems, the adsorption energies decrease with the increasing the atomic number of decoration atoms, that is, the largest adsorption energy of 0.831 eV for NO molecule on bP-Li and the smallest adsorption energy of 0.466 eV for NO molecule on bP-Cs. Similarly, the adsorption energies of NO molecules on bP-AEM are 1.538, 1.363 and 1.339 eV respectively, which decrease with increasing the atomic number of decoration atoms. On the other hand, from Table 1 we can see that the adsorption energies of NO molecule on bP-AEM are larger than those of NO molecule on bP-AM, indicating that the decoration of AEM are more effective than AM in improving the adsorption strength of phosphorene to NO molecules. The adsorption energies of NO on bP-Ni and bP-La are up to 2.121 and 2.475 eV, which are the largest adsorption energies. However, the NO molecule on bP-Tl has the smallest adsorption energy of 0.252 eV, which is even smaller than that on pristine phosphorene.

**Figure 6:**
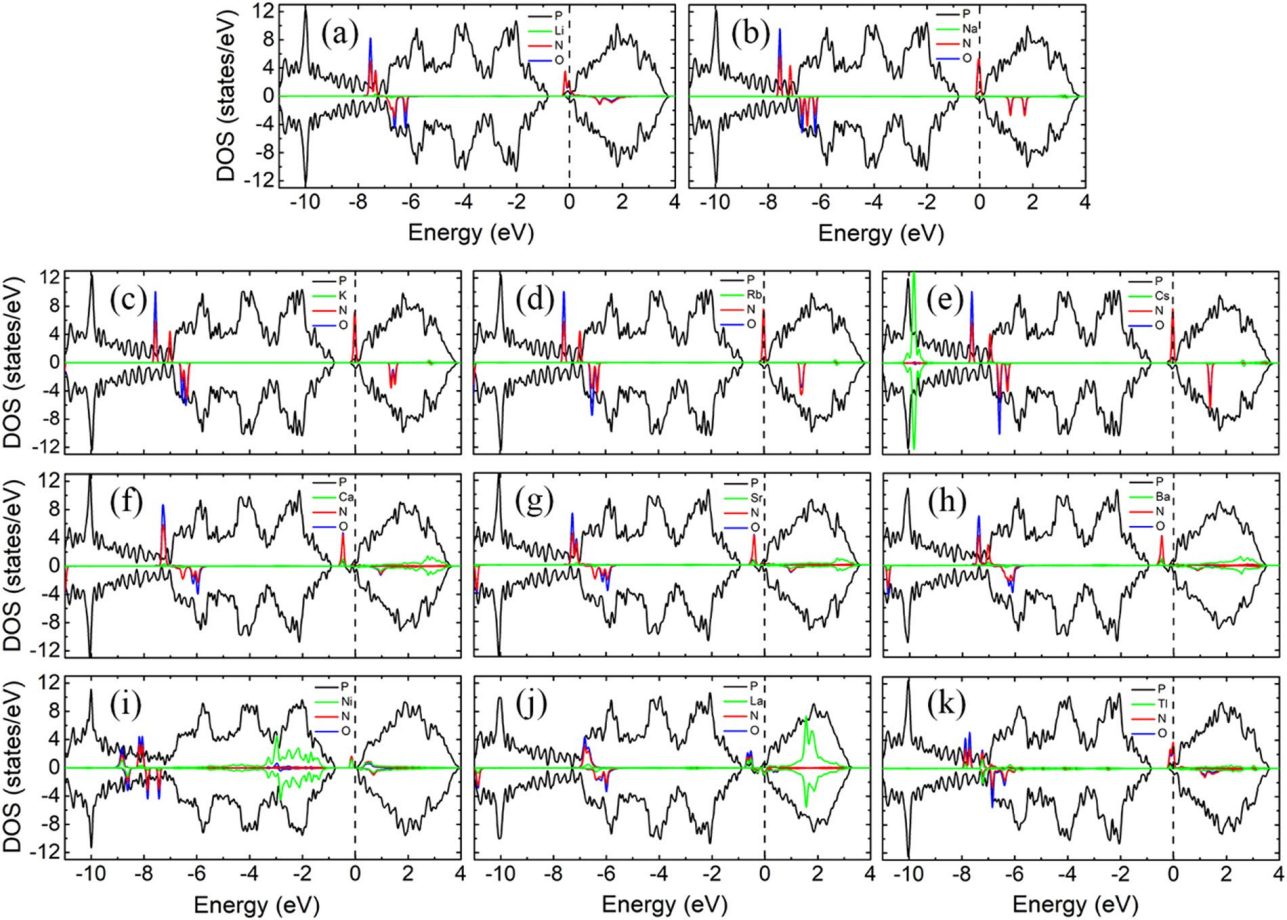
The LDOSs of NO adsorption on bP-M (M = Li, Na, K, Rb, Cs, Ca, Sr, Ba, Ni, La, Tl). The black, green, red and blue curves represent the LDOS of P, metal, N and O, respectively. The Fermi level is set to zero.

Figure 6 shows the LDOSs of NO molecule adsorptions on bP-M systems. The NO adsorption has large impact on the electronic properties of metal decorated phosphorene substrates. As shown in Fig. 6, all the LDOSs of adsorption systems exhibit asymmetric distribution, which means that spin polarizations present in all the bP-M systems. On the other words, except for bP-La system, the remaining bP-M systems undergo nonmagnetism-to-magnetism transition after NO adsorption. The magnetic moments of NO adsorbed bP-M systems are summarized in Table 1. Interestingly, except for the bP-La system, the adsorptions of NO molecules only introduce a significant spin-up peak in the bandgaps of all the other bP-M systems. The bP-La system has a large spin-up peak and a small spin-down peak, introduced by NO adsorption, in the bandgap.

From Fig. 6, we can see that the Fermi levels of the adsorption systems exhibit different degree of downward shifting due to the electrons transfer from the metal decorated phosphorene substrates to NO molecules. Although the impurity states are introduced into the bandgap of the bP-Ni system, the adsorption of NO molecule doesn’t change the conductive behavior of the bP-Ni system. That is, the bP-Ni system still remains the semiconductor property after NO adsorption. Furthermore, the band structure for NO adsorbed bP-Ni system in Fig. S8(i) is similar to that of the NO adsorbed on pristine phosphorene system, which indicates that metal decoration has little effect on the band structure of NO molecule adsorption on phosphorene. A slight upward shift of Fermi level for NO adsorbed on phosphorene is observed after Ni decoration, which may be ascribed to a small amount of electrons transfer from metal to phosphorene. The VBM of bP-Ni moves from Γ-point to Y-point after NO adsorption, and thus NO adsorbed bP-Ni system experiences direct-to-indirect transition with an indirect bandgap of 0.953 eV, which is larger than bandgap of 0.788 eV for without NO adsorption case. The increase of bandgap for the NO adsorbed bP-Ni system may be ascribed to charge transfer from bP-Ni substrate to NO molecule which results in the Ni states being lower the VBM of phosphorene. Except for the bP-Ni adsorption systems, the LDOSs of all the other bP-M adsorption systems at Fermi level are non-zero, implying that all the other bP-M adsorption systems are of metallic conductivities, which can also be demonstrated by Fig. S8. As compared with the bP-M systems without gas molecule adsorptions, the bP-Ca, bP-Sr, and bP-La adsorption systems experience semiconductor-to-metal transition after NO molecule adsorption, while the conductive properties of other bP-M adsorption systems remain unchanged. Furthermore, the lowest conduction bands of bP-M adsorption systems, especially for those of bP-AEM ones, are pushed up. The separations between the lowest and the second lowest conduction bands decrease, which may be due to that the repulsive interactions between the NO states above Fermi levels and the lowest conduction band of phosphorene are weakened by the interactions of NO molecule with metal atoms^43^.

As mentioned above, except for Tl decoration case, all the metal decoration can increase the adsorption energies of NO adsorption on phosphorene, and thus improve the adsorption of phosphorene to NO molecules. For the bP-AM adsorption system, the states of AM atoms distribute mainly in conduction bands as mentioned before, and thus the peaks, near VBM and in the conduction bands, from N and O atomic states hybridized with those of AM atoms. For the bP-AEM adsorption system, the spin-up states in bandgap from NO molecules overlap significantly with those from AEM atoms, which account for the larger adsorption energies of NO adsorbed bP-AEM systems. For the LDOSs of NO adsorpted bP-Ni as depicted in Fig. 6(i), the energy positions of NO LDOS peaks are almost unchanged as compared with the LDOSs of NO adsorbed pristine phosphorene except for slight shift due to electrons transfering between Ni and phosphorene. However, the hybridization of Ni LDOS peaks with NO LDOS peaks can be seen clearly in Fig. 6(i). Similarly, the strong hybridization of La LDOS peaks with NO LDOS peaks, especially the peaks near Fermi level, can be found in Fig. 6(j), which may be the cause of the largest adsorption energy.

### NO_2_ gas molecules adsorption on bP-M

**Figure 7:**
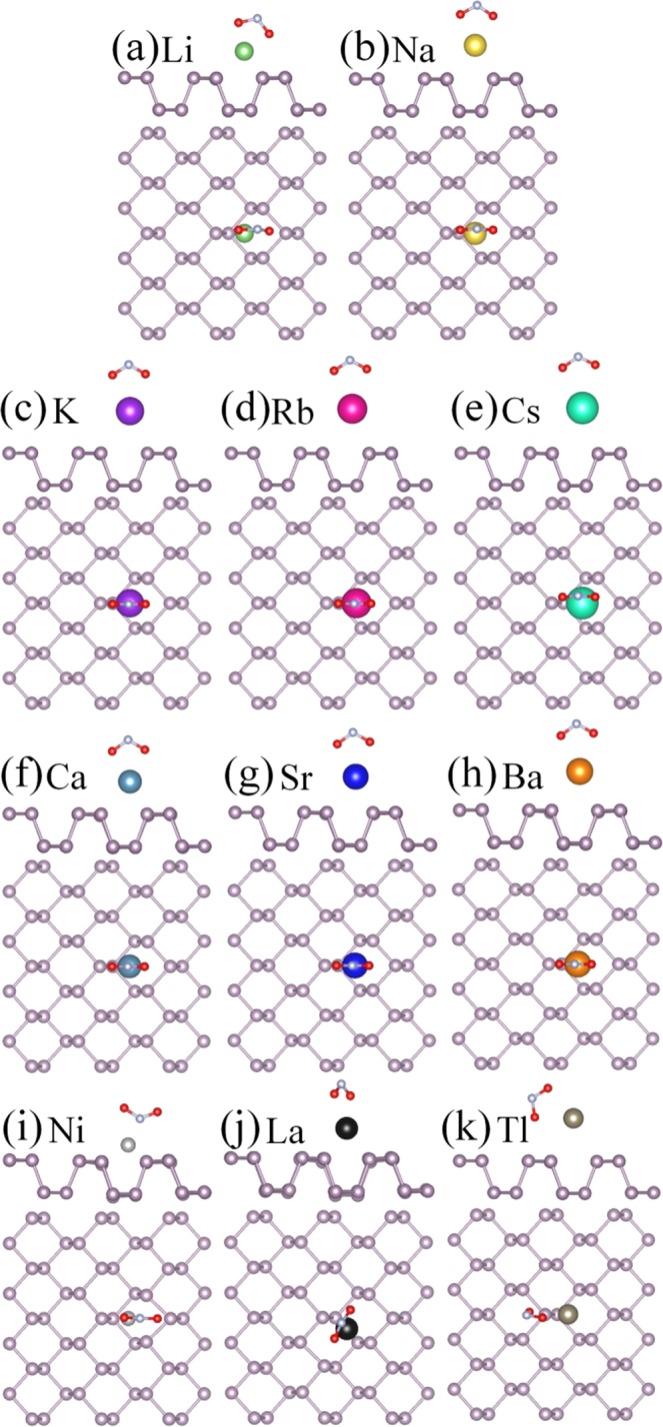
The optimized structures of NO_2_ adsorption on bP-M (M = Li, Na, K, Rb, Cs, Ca, Sr, Ba, Ni, La, Tl). The green, gold, purple, red, cyan, dark cyan, bule, orange, light gray, black and gray balls represent Li, Na, K, Rb, Cs, Ca, Sr, Ba, Ni, La and Tl, respectively.

Various initial adsorption configurations are considered and the most stable adsorption configurations are picked out, by comparing the adsorption energies of various initial configurations, as illustrated in Fig. 7. The adsorption energies for the most stable configurations are list in Table 1. The NO_2_ molecule is adsorbed on the bP-Ni system with the bond angle away from Ni atom, while the remaining NO_2_ molecules are stably adsorbed on the metal decorated substrates with the bond angles toward metal atoms. Similar to NO molecules, the adsorption energies of NO_2_ molecules on bP-AM and bP-AEM systems decrease with increasing the atomic number of decoration atoms, and the adsorption energies of NO_2_ on bP-AEM are larger than those on bP-AM (see Fig. S6 and Table 1). For transition metal Ni and La decorated phosphorene cases, the corresponding NO-molecule adsorption energies are 1.818 and 3.734 eV, respectively. The adsorption energy of NO_2_ molecule on bP-La is the largest among all adsorption energies. The adsorption energy of NO_2_ molecule on bP-Tl is only 1.773 eV.

**Figure 8:**
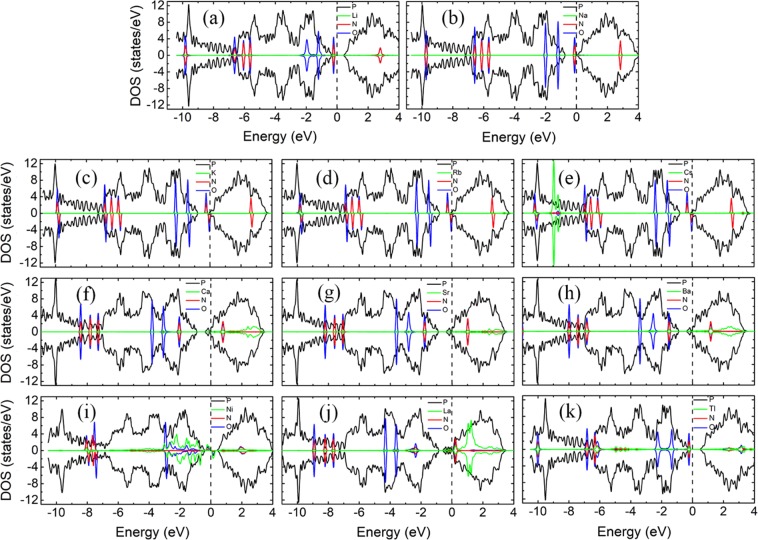
The LDOSs of NO_2_ adsorption on bP-M (M = Li, Na, K, Rb, Cs, Ca, Sr, Ba, Ni, La, Tl). The black, green, red and blue curves represent the LDOS of P, metal, N and O, respectively. The Fermi level is set to zero.

To better understand the effects of NO_2_ molecules on the bP-M systems, the LDOSs of NO_2_ molecule adsorbed bP-M systems are shown in Fig. 8. The LDOSs of bP-K, bP-Rb, bP-Cs and bP-Ni adsorption systems exhibit obvious asymmetry distribution, indicating that bP-K, bP-Rb, bP-Cs and bP-Ni are spin polarized with magnetic moment of 0.166, 0.192, 0.240 and 1.000 μB, respectively, as given in Table 1. The spin polarized character of bP-K, bP-Rb, bP-Cs and bP-Ni adsorption systems can also be corroborated by the band structures in Fig. S9. All the other NO_2_ adsorbed bP-M systems exhibit non-spin polarized characters. Especially for bP-La system, which is spin polarized before gas molecule adsorption, it exhibits symmetric distributed LDOS after NO_2_ adsorption, indicating that bP-La adsorption system is non-spin polarized with zero magnetic moments (see Table 1). Disappearance of the magnetism for bP-La adsorption system may be attributed that the spin polarization of the substrate is inhibited by the adsorption of NO_2_. As compared with bP-Ms without gas molecule adsorptions case, the bP-K, bP-Rb, bP-Cs and bP-Ni systems experience nonmagnetism-to-magnetism transition, while the bP-La system experiences magnetism-to-nonmagnetism transition, after NO_2_ adsorption.

From Table 1, we can see that the electrons transfer from bP-M substrates to NO_2_ molecules for all the NO_2_ adsorbed bP-M systems, which implies that the Fermi levels of all the bP-M systems shift down after NO_2_ molecule adsorptions. For bP-K, bP-Rb and bP-Cs adsorption systems, the N and O atoms introduce a spin-up peak in the bandgap and a spin-down peak at the CBM. The Fermi levels of bP-K, bP-Rb and bP-Cs shift to the CBM and cross through the spin-down states of N and O atoms, leading to the metal properties. For bP-Li and bP-Na systems, the N and O atoms introduce the symmetric states near VBM and CBM for bP-Li and bP-Na adsorption systems, respectively. The Fermi levels of bP-Li and bP-Na systems shift downward into the bandgaps of phosphorene after NO_2_ molecule adsorptions, indicating that the NO_2_ adsorbed bP-Li and bP-Na systems are semiconductors. The bandgaps of NO_2_ adsorbed bP-Li and bP-Na are 0.921 and 0.902 eV, respectively, which are slightly larger than that of pristine phosphorene. This may be ascribed to decrease of repulsive interaction between the facing lone pair electrons at channel due to the wider channel^42^. For bP-AEM systems, as shown in Figs 8(f–h) and S9(f–h), the separations between the lowest and the second lowest conduction bands significantly decrease due to that the adsorptions of NO_2_ molecules weaken the repulsive interaction between the Ni atom states and the lowest conduction band of phosphorene^42^. This leads to the lowest conduction bands shifting upward, and the Fermi levels cross through the lowest conduction bands. Thus, the NO_2_ adsorbed bP-Ca, bP-Sr and bP-Ba systems exhibit the metal properties. As shown in Fig. 8(i), the adsorption of NO_2_ weakens the interaction between Ni atom and the phosphorene, and thus the Ni atom states are away from VBM of phosphorene. This leads to the band of bP-Ni shifting slightly downward. Moreover, the VBM shifts from the Γ- to Y-point, which makes the bP-Ni adsorption system become into an indirect bandgap semiconductor. As shown in Fig. 8(j), compared with before NO_2_ adsorption, the impurity band of bP-La near the Fermi level disappears after NO_2_ adsorption. Thus, bP-La remains the small indirect-bandgap-semiconductor properties. In Fig. 8(k), similar to bP-Li and bP-Na systems, the adsorption of NO_2_ molecules causes the Fermi level to shift from the conduction band significantly downward into the bandgap. Thus, bP-Tl exhibits semiconductor properties after NO_2_ adsorption, and the corresponding bandgap is 0.924 eV. Therefore, after NO_2_ adsorption, the bP-Li, bP-Na, and bP-Tl systems undergo metal-to-semiconductor transition, while the bP-Ca and bP-Sr systems undergo semiconductor-to-metal transition. Additionally, the bP-Ni system remains semiconductor property, but it undergoes direct-to-indirect transition after NO_2_ adsorption.

The coupling peaks between the NO_2_ molecules and metals of NO_2_ adsorpted bP-AEM systems are more widely distributed, especially in the conduction band, than those of NO_2_ adsorbed bP-AM systems, which may account for that the AEM decoration is more effective in improving the adsorption strength of NO_2_ gas molecules on phosphorene. For bP-La adsorption system, the strong coupling peaks between the NO_2_ molecules and metal are observed around Fermi level, indicating a strong electronic interaction of the NO_2_ gas molecule with metal and thus the largest adsorption energy as shown in Table 1.

## Conclusions

In conclusion, the nitrogen-based gas molecules (NH_3_, NO, NO_2_) adsorption on metal (Li, Na, K, Rb, Cs, Ca, Sr, Ba, Ni, La, Tl) decorated phosphorene has been studied by using first-principles calculation based on DFT. Except for NO adsorption on bP-Tl, all metal decorations can improve the adsorption strength of phosphorene to nitrogen-based gas molecules. For NH_3_ and NO, the adsorption energies follow an energy order of *E*_*ads*_ (bP-TM) > *E*_*ads*_ (bP-AEM) > *E*_*ads*_ (bP-AM) > *E*_*ads*_ (bP-Tl), while the NO_2_ follow an energy order of *E*_*ads*_ (bP-La) > *E*_*ads*_ (bP-AEM) > *E*_*ads*_ (bP-AM). The largest energies of NH_3_, NO and NO_2_ adsorbed bP-M are 1.305, 2.475 and 3.734 eV, respectively. In addition, the band structures and LDOSs show that NH_3_ has little effect on electronic and magnetic properties of bP-M substrates, while the adsorptions of NO and NO_2_ make an interesting change in electronic and magnetic properties of bP-M. For example, except for bP-La, the metal decorated substrates exhibit nonmagnetism-to-magnetism after NO adsorption, and the bP-Ca, bP-Sr and bP-La undergo semiconductor-to-metal transition. Similarly, the magnetic states of some decorated substrates (bP-K, bP-Rb, bP-Cs, bP-Ni, bP-La) change after NO_2_ adsorption, and especially the spin polarization for bP-La systems disappear after NO_2_ adsorption. Furthermore, the bP-Li, bP-Na and bP-Tl experience metal-to-semiconductor transition, while bP-Ca and bP-Sr undergo semiconductor-to-metal transition due to large electron transfer amounts between NO_2_ and decorated substrates. These results indicate that the metal decoration can be used for the removal and detection of nitrogen-based gas.

## Supplementary information

Supporting Information
