# Supplementary material for: Nitrogen-based gas molecule adsorption of monolayer phosphorene under metal functionalization
Source: Sci Rep. 2019 Aug 29;9:12498. doi: 10.1038/s41598-019-48953-0 (PMC6715726; doi:10.1038/s41598-019-48953-0)
Supplement: Supplementary file 1 — Supporting Information [file 41598_2019_48953_MOESM1_ESM.docx]

Supporting Information

**Nitrogen-based gas molecule adsorption of monolayer** **phosphorene under metal functionalization**

Shuangying Lei^a,^*, Ran Gao^a^, Xiaolong Sun^a^, Sijia Guo^a^, Hong Yu^a^, Neng Wan^a^, Feng Xu^a^, Jie Chen^a^

^a^ Key Laboratory of Microelectro mechanical Systems of the Ministry of Education, Southeast University, Nanjing 210096, China.

*Corresponding author. E-mail address: lsy@seu.edu.cn (S. Lei).


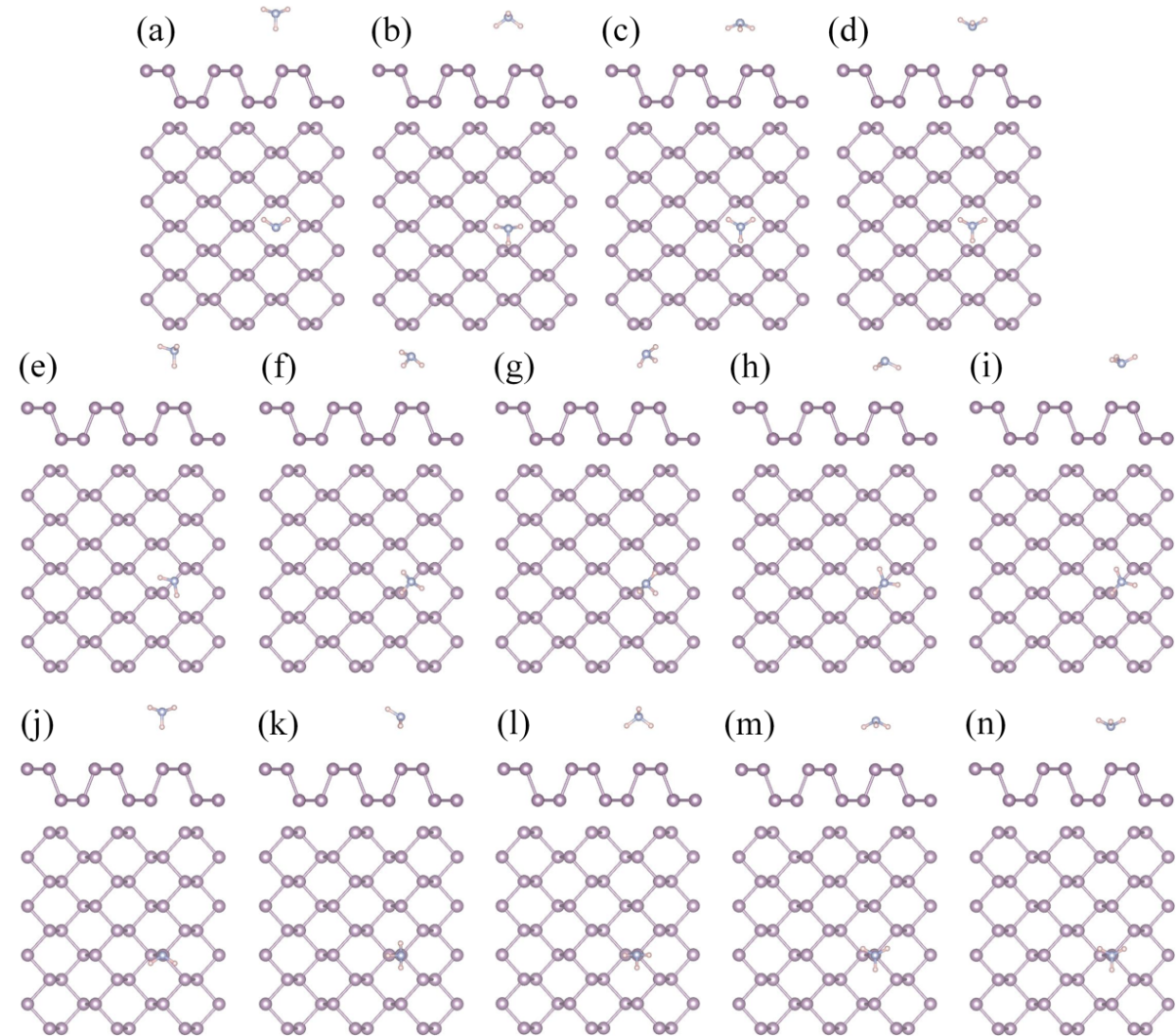


**Figure S1.** The initial structures of NH_3_ absorbed on 3×4 pristine phosphorene in different adsorption sites, N atom on hollow (a) one N-H bond vertical phosphorus plane, two H atom upwards (b) one H-H bond parallel phosphorus plane, one H atom upwards, (c) three H atoms downwards, (d) three H atoms upwards; N atom on bridge (e) one N-H bond vertical phosphorus plane, two H atom upwards, (f) one H-H bond parallel phosphorus plane, one H atom upwards, (g) one H atoms upwards, two H atoms downwards, (h) three H atoms downwards, (i) three H atoms upwards; N atom on top (j) one N-H bond vertical phosphorus plane, two H atom upwards, (k), (l) one H-H bond parallel phosphorus plane with H towards different direction (m) three H atoms downwards, (n) three H atoms upwards. Purple, gray and pink balls represent P, N and H atoms, respectively.


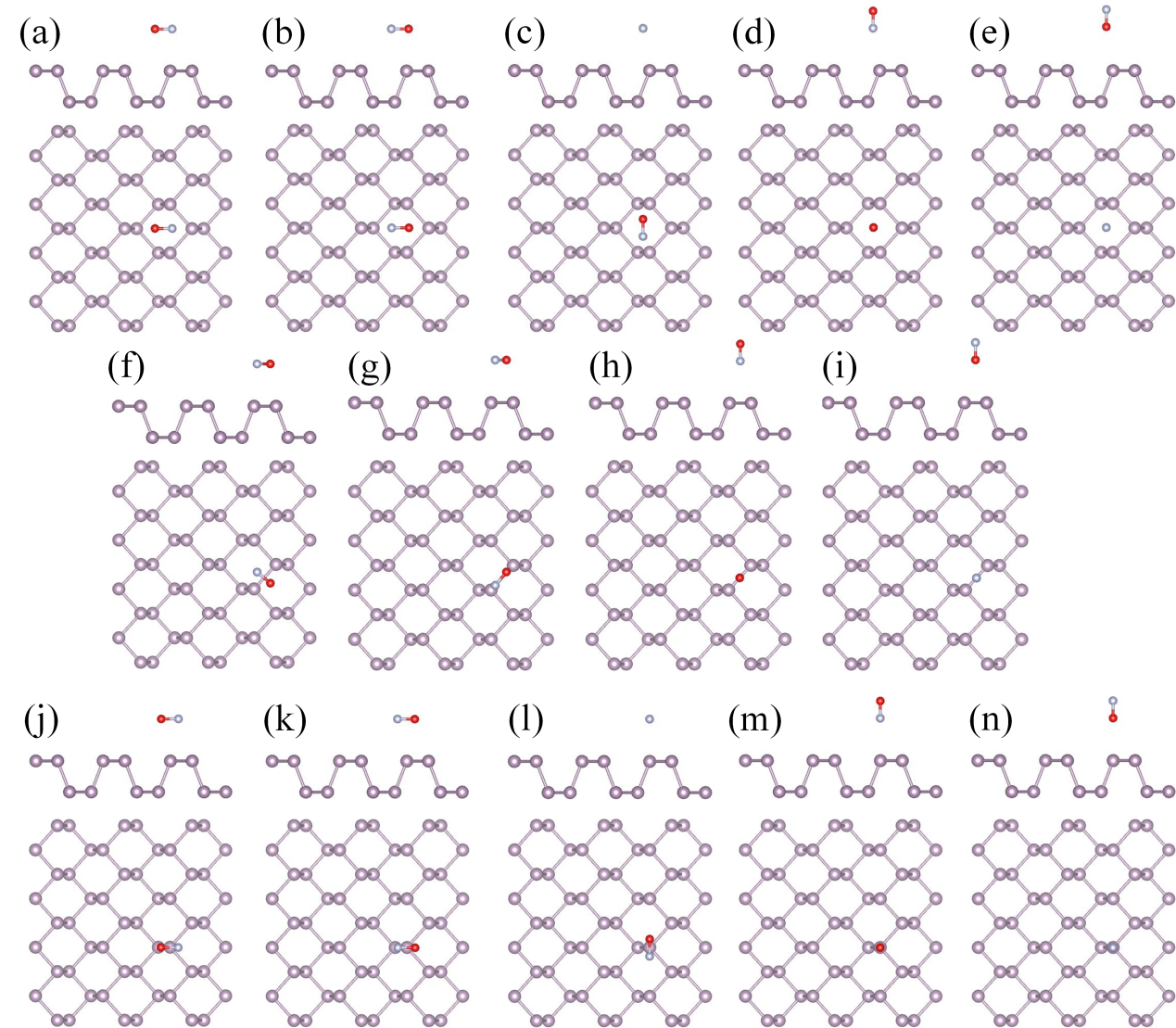


**Figure S2.** The initial structures of NO absorbed on 3×4 pristine phosphorene in different adsorption sites, N atom on hollow (a)-(c), (f) N-O bond parallel phosphorus plane with N towards different directions, (d) N-O bond vertical phosphorus plane with N downwards, (e) N-O bond vertical phosphorus plane with N upwards; N atom on bridge (g) N-O bond parallel phosphorus plane, (h) N-O bond vertical phosphorus plane with N downwards, (i) N-O bond vertical phosphorus plane with N upwards; N atom on bridge (j)-(l) N-O bond parallel phosphorus plane with N towards different directions, (m) N-O bond vertical phosphorus plane with N downwards, (n) N-O bond vertical phosphorus plane with N upwards. Purple, grey and red balls represent P, N, O atoms, respectively.


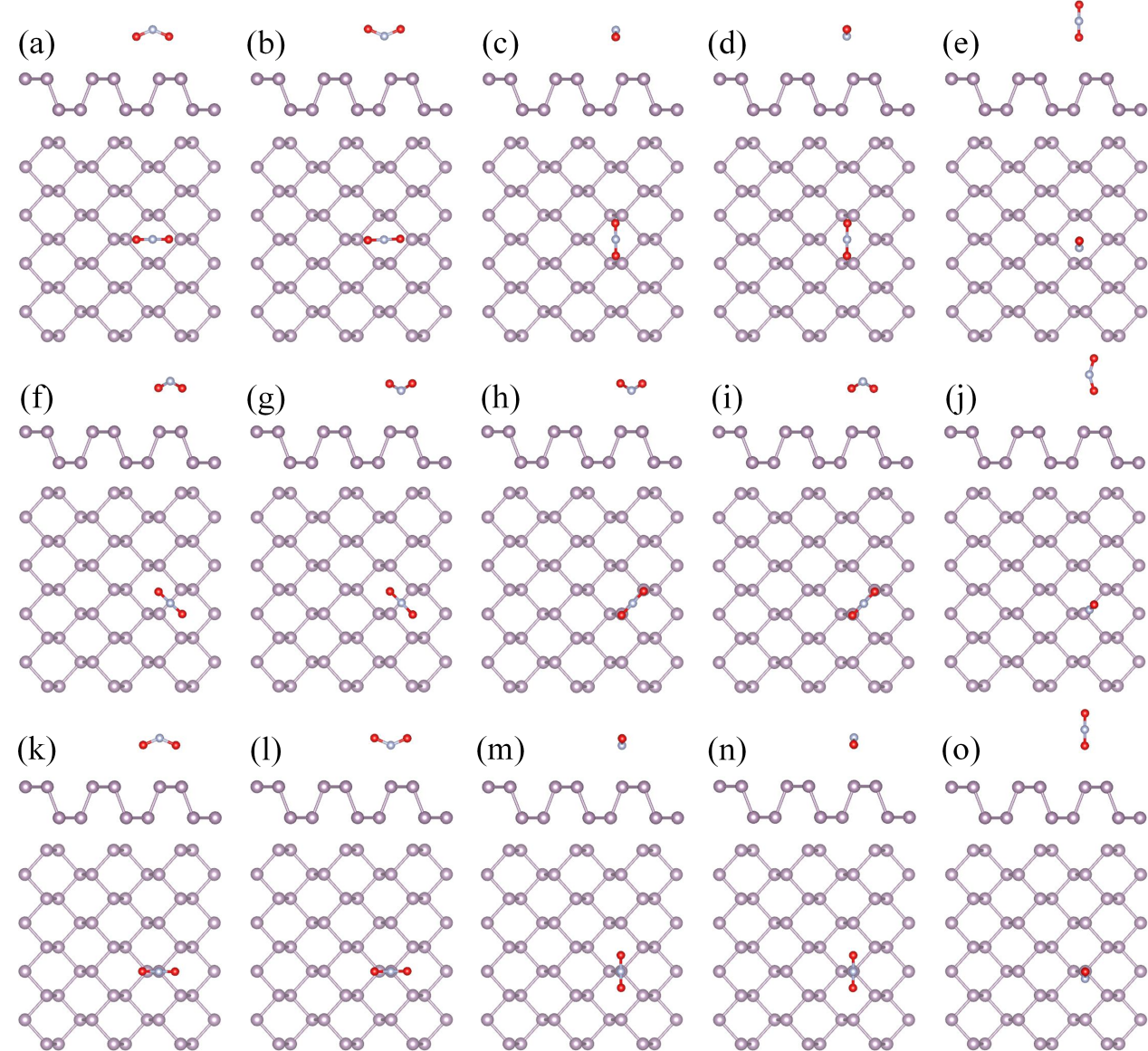


**Figure S3.** The initial structures of NO_2_ absorbed on 3×4 pristine phosphorene in different adsorption sites, N atom on hollow (a)-(d) H-H bond parallel phosphorus plane with H towards different directions, (e) H-H bond vertical phosphorus plane; N atom on bridge (f)-(i) H-H bond parallel phosphorus plane with H towards different directions, (j) H-H bond vertical phosphorus plane; N atom on top (k)-(n) H-H bond parallel phosphorus plane with H towards different directions, (o) H-H bond vertical phosphorus plane. Purple, grey and red balls represent P, N, O atoms, respectively.


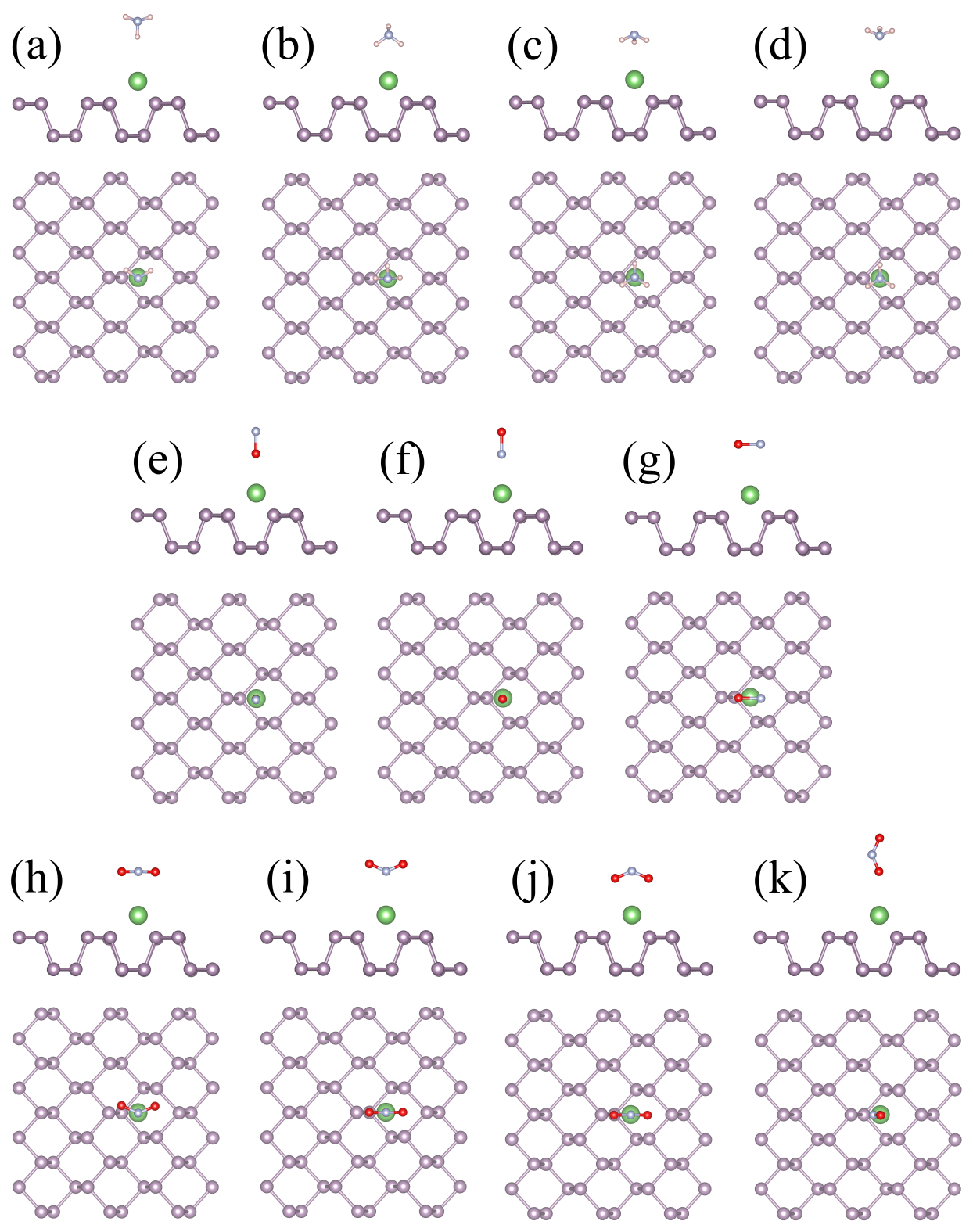


**Figure S4.** The initial structures of (a)-(d) NH_3_, (e)-(g) NO, (h)-(k) NO_2_ absorbed on Li doped phosphorene. Purple, grey, red and green balls represent P, N, O and Li atoms, respectively.


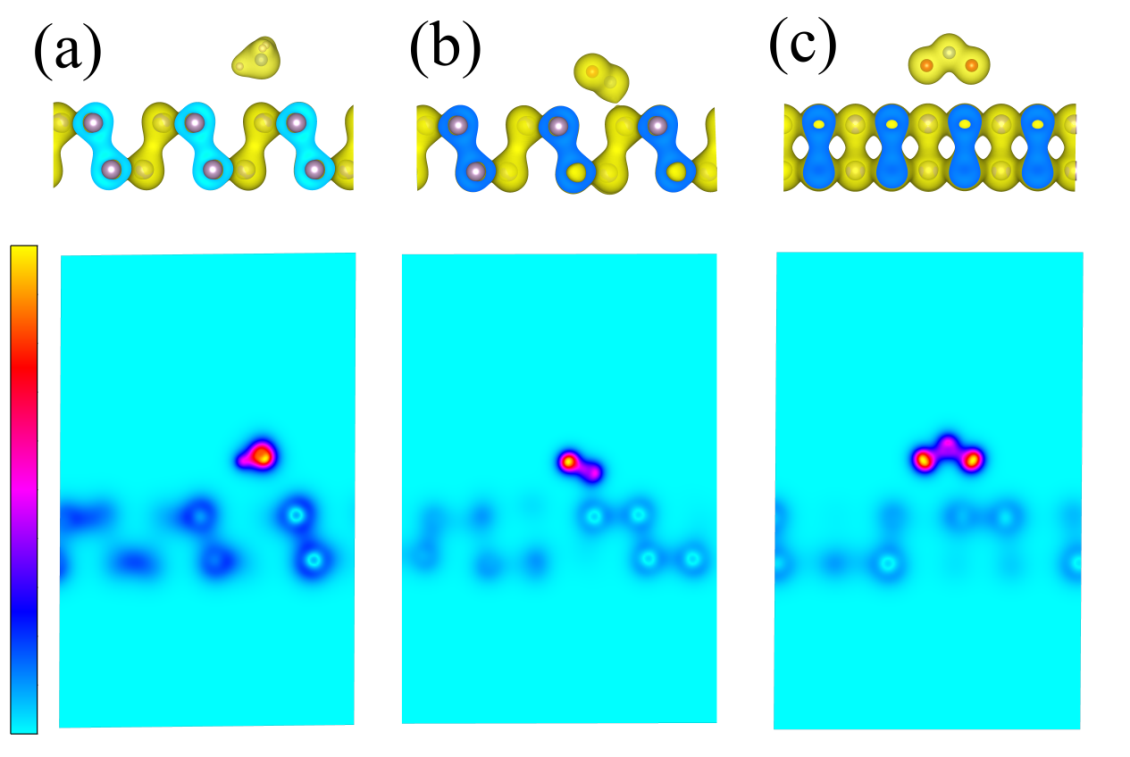


**Figure S5.** The charge densities (upper panels) and the slices of charge densities (lower panels) of (a) NH_3_, (b) NO and (c) NO_2_ adsorbed on phosphorene.


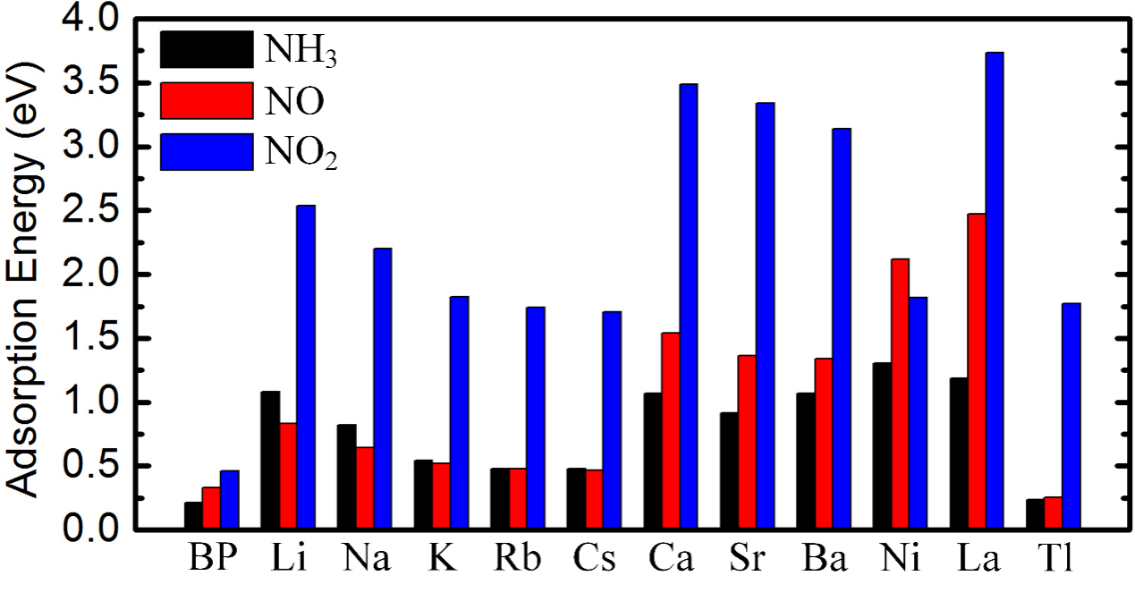


**Figure S6.** The adsorption energies of nitrogen-based gas molecules on bP-M.


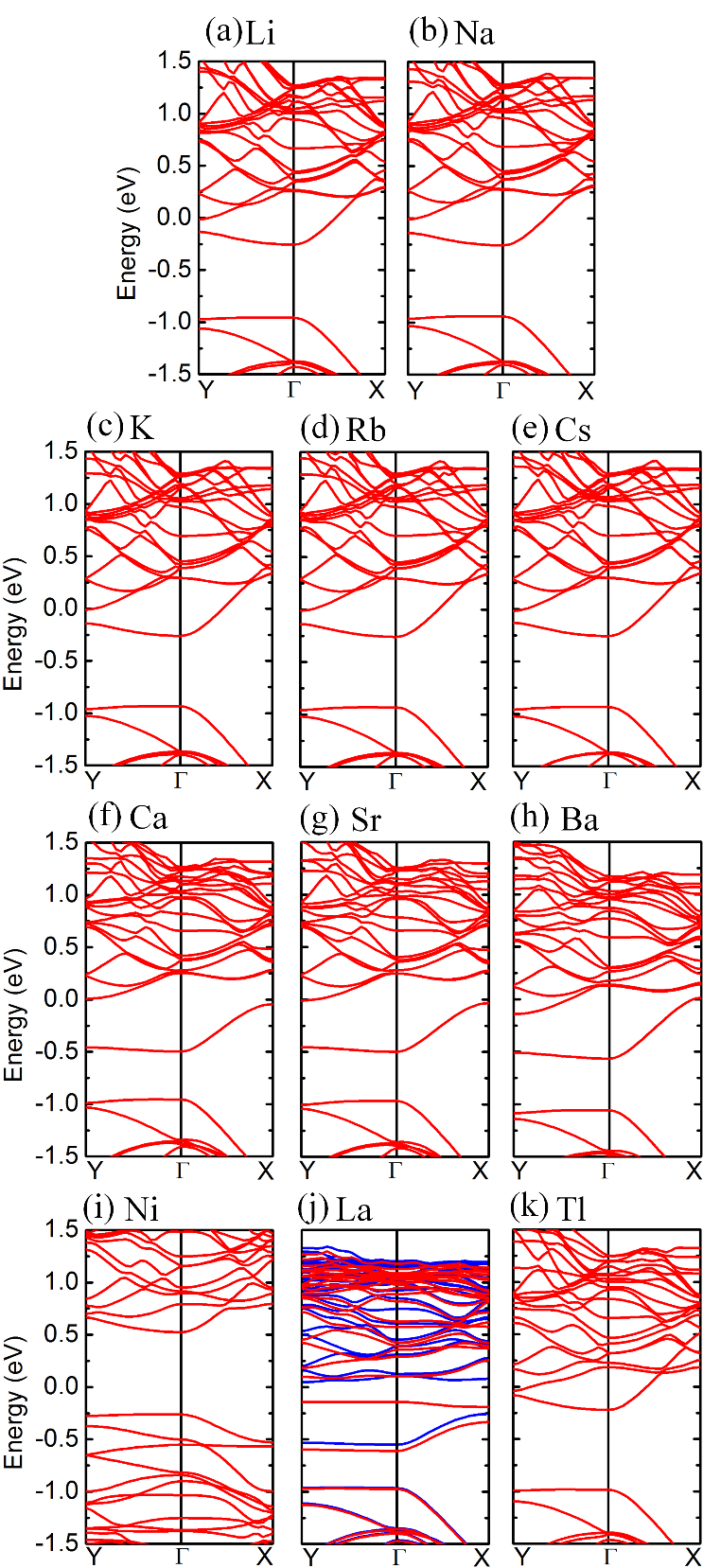


**Figure S7.** The band structures of NH_3_ adsorption on bP-M (M=Li, Na, K, Rb, Cs, Ca, Sr, Ba, Ni, La, Tl). The red and blue curves represent the spin-up and spin-down bands, respectively. The Fermi level is set to zero.


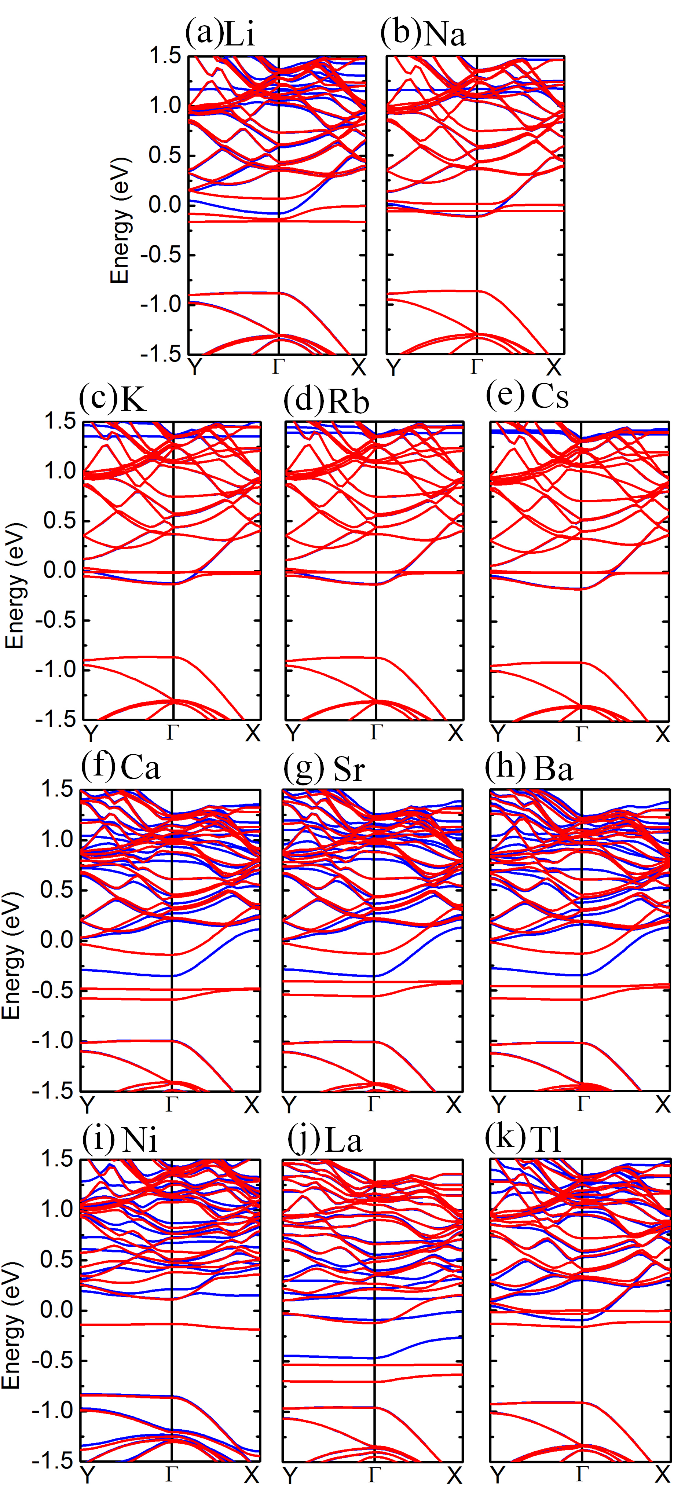


**Figure S8.** The band structures of NO adsorption on bP-M (M=Li, Na, K, Rb, Cs, Ca, Sr, Ba, Ni, La, Tl). The red and blue curves represent the spin-up and spin-down bands, respectively. The Fermi level is set to zero.


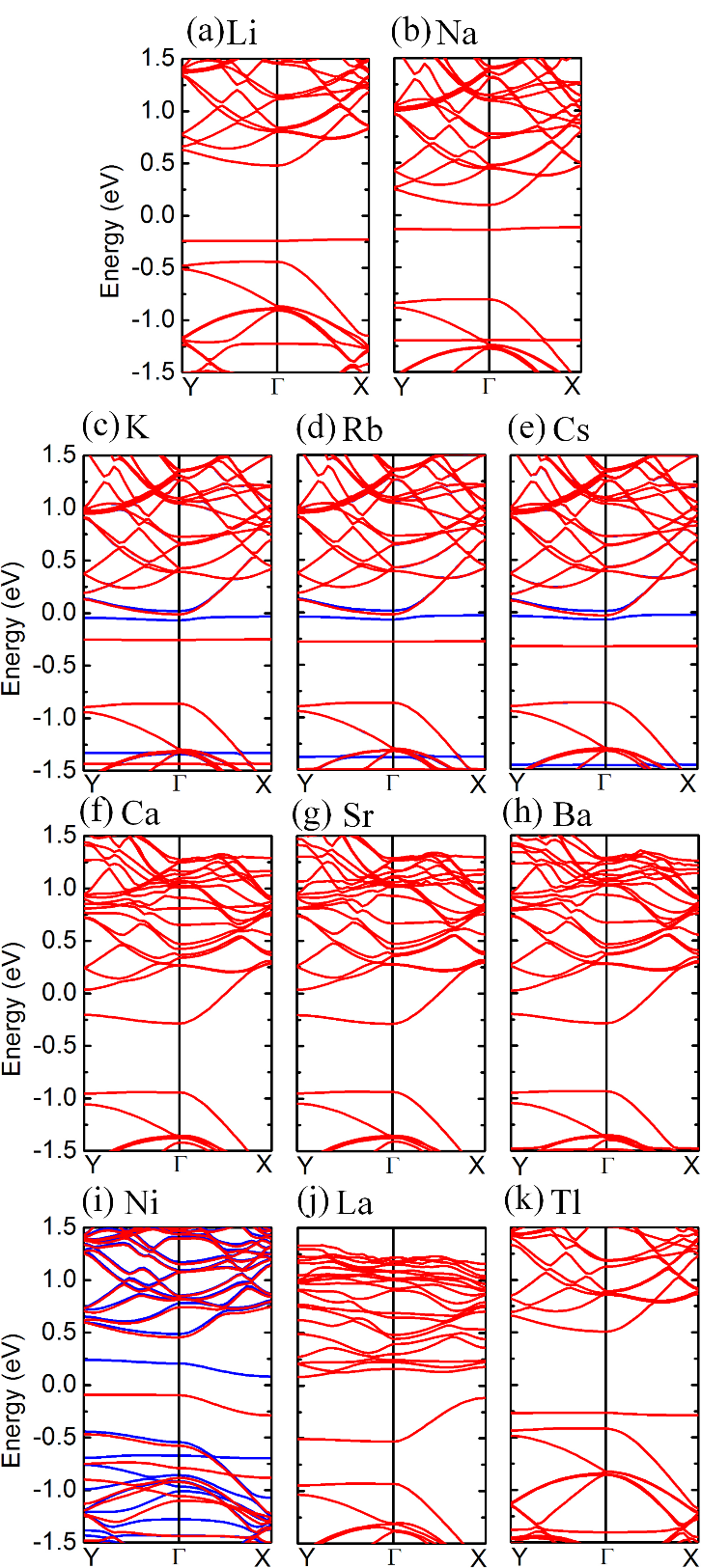


**Figure S9.** The band structures of NO_2_ adsorption on bP-M (M=Li, Na, K, Rb, Cs, Ca, Sr, Ba, Ni, La, Tl). The red and blue curves represent the spin-up and spin-down bands, respectively. The Fermi level is set to zero.
